# TREM1 Blockade Ameliorates Lipopolysaccharide-Induced Acute Intestinal Dysfunction through Inhibiting Intestinal Apoptosis and Inflammation Response

**DOI:** 10.1155/2021/6635452

**Published:** 2021-04-16

**Authors:** Lijuan Shen, Yonghua Zhou, Xiping Wu, Yuewen Sun, Tao Xiao, Yin Gao, Jingui Wang

**Affiliations:** ^1^Wuxi Hospital of Traditional Chinese Medicine, Nanjing University of Chinese Medicine Affiliated Wuxi Hospital, Wuxi, 214071 Jiangsu, China; ^2^Jiangsu Institute of Parasitic Diseases, Key Laboratory on Technology for Parasitic Disease Prevention and Control, Ministry of Health, Jiangsu Provincial Key Laboratory on Molecular Biology of Parasites, Jiangsu Provincial Key Subject on Parasitic Diseases, Wuxi 214064, China

## Abstract

**Objective:**

The lipopolysaccharide- (LPS-) induced acute intestinal dysfunction model has been widely applied in recent years. Here, our aim was to investigate the effect of triggering receptor expressed on myeloid cells-1 (TREM1) inhibitor in LPS-induced acute intestinal dysfunction.

**Methods:**

Male rats were randomly assigned into normal (saline injection), model (LPS and saline injection), and LP17 (LPS and LP17 (a synthetic TREM1 inhibitor) injection) groups. The levels of intestinal TREM1 expression were evaluated by immunohistochemistry and western blot. Intestinal permeability and apoptosis were separately assessed by the lactulose/mannitol (L/M) ratio and TUNEL assay. The levels of soluble TREM1 (sTREM1), TNF-*α*, IL-6, and IL-1*β* were measured in the plasma and intestinal tissues by ELISA. The expression levels of NF-*κ*B, high-mobility group box 1 (HMGB1), and toll-like receptor 4 (TLR-4) were measured with RT-qPCR and western blot. After transfection with si-TREM1 in LPS-induced intestinal epithelium-6 (IEC-6) cells, p-p65 and p-I*κ*B*α* levels were detected by western blot.

**Results:**

LP17-mediated TREM1 inhibition alleviated the intestine tissue damage in rats with LPS-induced acute intestinal dysfunction. LP17 attenuated the LPS-induced increase in sTREM1, TNF-*α*, IL-6, and IL-1*β* levels in the plasma and intestinal tissues. Furthermore, intestine permeability and epithelial cell apoptosis were ameliorated by LP17. LP17 attenuated the LPS-induced increase in the expression of TREM1, HMGB1, TLR-4, and NF-*κ*B in the intestine tissues. In vitro, TREM1 knockdown inactivated the NF-*κ*B signaling in LPS-induced IEC-6 cells.

**Conclusion:**

LP17 could ameliorate LPS-induced acute intestinal dysfunction, which was associated with inhibition of intestinal apoptosis and inflammation response.

## 1. Introduction

Intestinal inflammation is the host's invasion defense response through microbial toxins (such as lipopolysaccharide (LPS)) or pathogens (such as Escherichia coli) [1], which is a key risk factor for highly fatal diseases like colorectal cancer [2]. Immunity imbalance and mucosal barrier destruction are the main mechanisms of intestinal inflammation [3]. Inflammation may thin the mucosal layer, reduce the lumen coverage and the adhesion, and destroy the integrity of the intestine by increasing the apoptosis and permeability of epithelial cells and inhibiting cell proliferation, inducing the loss of barrier function, and ultimately leading to acute intestinal dysfunction [4–6]. Furthermore, acute intestinal dysfunction triggers the spread of metabolites, endotoxins, and bacteria, an effect caused by enterogenous bacteremia, thereby exacerbating sepsis and multiple organ dysfunction syndrome (MODS) [4–6]. Previous studies on biomarkers of intestinal failure have demonstrated that the disease severity and intensive care unit mortality have both been linked to elevated expression of intestinal fatty acid-binding protein (I-FABP, a biomarker of enterocyte damage), decreased expression of citrulline (a biomarker of enterocyte mass and damage), and increased intestinal permeability [7, 8]. Although our understanding on the pathophysiology of acute intestinal dysfunction has been greatly improved [9], the treatment of acute intestinal dysfunction is restricted to proton-pump inhibitors, antibiotics, and parenteral nutrition [10]. So far, no targeted therapeutics for acute intestinal dysfunction have been approved for clinical use [9].

Triggering receptor expressed on myeloid cells-1 (TREM1) belongs to a family of innate immune receptors, which is mainly expressed on murine and human granulocytes and monocytes/macrophages [11]. TREM1 can synergize with various toll-like receptors (TLRs) and trigger nuclear factor- (NF-) *κ*B signaling to induce the expression of proinflammatory cytokines, such as tumor necrosis factor- (TNF-) *α*, interleukin- (IL-) 6, and IL-1*β*, thereby amplifying inflammatory response [12–15]. Furthermore, soluble TREM1 (sTREM1) has been proposed as a diagnostic and prognostic biomarker for patients with septic shock [16]. It has been shown that TREM1 is a promising diagnostic biomarker and therapeutic target for sepsis [17]. However, TREM1 expression is absent in myeloid cells of the normal intestine, which is a key mechanism for the prevention of intestinal inflammation and excessive tissue damage [18].

As in previous studies, it has been confirmed that TREM1 participates in inflammatory bowel diseases [19]. It promotes the progression of inflammatory bowel disease by enhancing proinflammatory response [20]. Blocking TREM1 expression can protect mice from intestinal ischemia-reperfusion injury [21]. Furthermore, TREM1 induces intestinal barrier dysfunction associated with pancreatitis [22]. Thus, TREM1 may play a key role in acute intestinal dysfunction. LPS is the main component of the cell wall of Gram-negative bacteria, which plays an important role in mediating intestinal inflammatory response [23]. Recent studies have shown that physiologically relevant LPS concentrations (0 to 1 ng/ml) can increase intestinal epithelial TJ permeability [24]. The LPS injury model has been widely applied in studying the intestinal TJ barrier. It has been reported that LP17, a synthetic TREM1 inhibitor, has a protective effect on LPS-induced lung injury [25] and can be used to treat mice with LPS-induced septic shock [26]. However, it remains unclear whether LP17 could protect against LPS-induced acute intestinal dysfunction and the related molecular mechanisms.

The LPS-induced acute intestinal dysfunction model has been widely used in recent years. For example, based on the LPS-induced intestinal injury model, it has been found that Rhein could ameliorate inflammatory and oxidative responses via modulating Nrf2 and MAPKs [27]. Furthermore, proanthocyanidins ameliorate inflammatory response and intestinal permeability in an LPS-induced intestinal injury rat model [28]. Thus, it is rational to use the intestinal injury model induced with LPS. In this study, we hypothesized that TREM1 inhibitor LP17 could ameliorate intestinal apoptosis and inflammatory response in LPS-induced acute intestinal dysfunction rats. Furthermore, we analyzed its relevant molecular mechanisms.

## 2. Materials and Methods

### 2.1. Animals and LPS-Induced Acute Intestinal Dysfunction Model

Sixty adult male Sprague Dawley (SD) rats (weighing 260–280 g; eight weeks old) were purchased from the specific pathogen free (SPF) Laboratory of the Animal Laboratory Center at the Key Laboratory on Technology for Parasitic Disease Prevention and Control, Ministry of Health (Wuxi City, China). All rats were maintained at 22 ± 1°C room temperature with a 12/12 h light/dark cycle and had free access to food and water. These rats were randomly assigned into three groups: normal group, LPS-induced acute intestinal dysfunction group, and LP17 group. The LPS-induced acute intestinal dysfunction model was established by intraperitoneal injection of 4.5 mg/kg LPS, as previously reported [29]. LP17 peptide (LQVTDSGLYRCVIYHPP) was synthesized by Shanghai Science Peptide Biological Technology Co., Ltd. (Shanghai, China). Its molecular weight was 2074.41, and purity was 98.22% that was verified by mass spectrometry and high-performance liquid chromatography (HPLC). LP17 synthetic peptide (3.5 mg/kg body weight [11]; Sigma, St. Louis, MO, USA) was administered to the LPS-induced acute intestinal dysfunction rats in the LP17 group through the vena caudalis at the time of LPS injection, while the rats in the normal and model groups were injected with an equal amount of saline. All rats were anesthetized by intraperitoneal injection of sodium pentobarbital (200 mg/kg body weight) and then euthanized with CO_2_ inhalation at 108 h after LPS administration. This animal experiment was approved by the Animal Care and Use Committee at Wuxi Hospital of Traditional Chinese Medicine (Wuxi, City, China). Our animal experimental procedures were conducted following the Guide for the Care and Use of Laboratory Animals.

### 2.2. Specimen Collection

72 h after LPS administration, all rats were administered by 2 ml isomolar solution containing 100 mg lactulose and 50 mg mannitol by intragastric administration, as previously described [30]. Urine samples were collected from each animal in a metabolic cage for over 24 h. Subsequently, 2.5 ml urine was stored in a smaller bottle containing 0.6 mg thimerosal to avoid bacterial growth. Blood samples were obtained from the orbital venous plexus 12 h later. After centrifuging the blood samples at 2000 × g for 10 min, the serum was stored at –80°C. After euthanizing the rats, small intestine tissues including intestinal feces were collected and frozen rapidly in liquid nitrogen and stored at −80°C for immunohistochemistry and pathological examination.

### 2.3. Enzyme-Linked Immunosorbent Assay (ELISA)

ELISA was used to determine the levels of sTREM1, TNF-*α*, IL-6, I-FABP, and citrulline [31] in the serum and cell supernatant using commercial kits (all from Shanghai Hufeng Chemical Co., Ltd., Shanghai, China) according to the manufacturer's protocols. The absorbance (optical density, OD) of each well was measured at a wavelength of 450 nm with a BioTek ELx800 microplate reader (Winooski, VT, USA).

### 2.4. HPLC Analysis of Intestinal Permeability

A lactulose/mannitol (L/M) ratio > 0.03 was used to define an increase in intestinal permeability. A L/M ratio > 0.10 was defined as a marked increase in intestinal permeability [30]. The urine samples were examined by HPLC using a Dionex Carbon-Pac PA1 ion exchange column at 30°C and equipped with a pulsed amperometric detector integrator using an Au main electrode and an AgCl reference electrode, and a water and NaOH gradient mobile phase [32]. A 1525 HPLC device (Waters, Milford, MA, USA) equipped with a 2489 UV detector (5 *μ*m, 4.6 × 250 mm, XBridge C18 column) was used for the metabolite analyses, and the device was operated by an Empower2 chromatography workstation.

### 2.5. Hematoxylin-Eosin (H&E) Staining of Small Intestine Tissues

The small intestine tissues were fixed in 10% formalin, dehydrated, paraffin-embedded, and sectioned. H&E staining of the sections was performed as previously described [33]. Under microscopy, the pathological changes of villi, intestinal layers, and epithelial cells were assessed by experienced pathologists. The DX45 software (DP2-BSW image analysis system, Olympus, Tokyo, Japan) was used to analyze the length of the villi and the mucosal thickness. Blinded procedures were ensured for the examiners by performing intergroup analysis.

### 2.6. Pathological Examination with Chiu Score

The six-point Chiu score was used to assess the intestinal mucosal damage based on a scale ranging from 0 to 5, with the pathological degree spanning from mild to severe [34]. The scoring system was defined as follows: 0, the normal villi of the small intestinal mucosa with no noticeable lesions; 1, the occurrence of Gruenhagen's space under the intestinal mucosa in the middle axis of villi, which was often accompanied by capillary congestion; 2, the elevation of intestinal mucosa epithelium from the lamina propria and the expansion of the upper and subcutaneous space of the intestine; 3, the elevation of large intestinal mucosa epithelium with the villi falling to both sides and villi top missing; 4, the shedding of villi and lamina propria, and the expansion of exposed capillaries, in which the cell composition of the lamina propria was increased; 5, the degeneration or digestion of the lamina propria, and bleeding or ulcer formation.

### 2.7. Immunohistochemistry

Paraffin-embedded tissues were cryosectioned into 4 *μ*m thickness, which were mounted on microscope slides with a charged surface. The UltraVision polymer detection method was used to determine the protein expression as previously described [35–37]. All the procedures for immunohistochemistry were based on the protocol of the commercial kit (Beijing Biosynthesis Biotechnology Co., Ltd., Beijing, China). The sections were baked under 65°C for 10 min and then boiled in a citrate buffer (pH = 6) for 30 min. Immunoblocking of nonspecific background was applied to the sections by incubating the sections in a hydrogen peroxide block for 10 min at room temperature. The sections were incubated with rabbit polyclonal antibodies (1 : 500; Beijing Biosynthesis Biotechnology Co., Ltd.) at room temperature for 2 h and goat anti-rabbit IgG (1 : 5000; Beijing Biosynthesis Biotechnology Co., Ltd.) for 30 min at room temperature. After washing with running tap water, the sections were treated with DAB brown solution (1 : 20) for 1 min and washed with running tap water for 2 min. After immunostaining, the slides were mounted and digitized with a ScanScope CS2 digital pathological scanning system (Leica Biosystems, USA). The semiquantitative analysis was presented via scoring the percentage of positive cells and staining intensity. The number of positive cells was as follows: <5% was 0 points, 5%-25% was 1 point, 26%-50% was 2 points, 51%-75% was 3 points, and 76%-100% was 4 points. Positive cell staining intensity scores were as follows: 0 for colorless, 1 for light yellow, 2 for brown, and 3 for tan. The product of the number of positive cells and the positive cell staining intensity was calculated, as follows: 0 for negative, 1-4 for weakly positive, 5-8 for positive, and 9-12 for strong positive.

### 2.8. Terminal Deoxynucleotidyl Transferase dUTP Nick-End Labeling (TUNEL) Staining

Apoptotic cells in the intestinal epithelium were detected by the TUNEL assay using the DeadEndTM Fluorometric TUNEL System (Promega, Madison, WI, USA) according to the manufacturer's protocol. Briefly, after deparaffinization with proteinase K digestion and incubation with 3% H_2_O_2_ blocking solution, the sections were titrated with 0.55 *μ*l/cm^2^ TdT enzyme and incubated at room temperature for 1 h. Then, the stopping solution was added to the sections, which were subsequently washed with phosphate-buffered saline (PBS) solution 4 times, 2 min for each wash. After titrated with DAB coloration solution, the sections were counterstained with 0.5% Brilliant Green and mounted with glycerol buffer. Three fields of view were randomly selected under a high-power field (×200). The number of positive cells was calculated in each field.

### 2.9. Cell Culture and Transfection

Intestinal epithelium-6 (IEC-6) cells were purchased from (Tongpai Biotechnology Co., Ltd., Shanghai, China) and maintained in Dulbecco's modified Eagle's medium supplemented with 10% fetal bovine serum (FBS; HyClone, Logan, UT, USA), 100 units/ml of penicillin, and 100 *μ*g/ml of streptomycin (Gibco) [38]. Cells were grown in a humidified atmosphere with 5% CO_2_ at 37°C. To mimic intestinal inflammation, IEC-6 cells were seeded into 24-well plates (5 × 104 cells/well) and cultured in the medium with 200 ng/ml LPS. When the cells reached a confluence of 70%-90%, siRNA oligos were transfected into the cells via lipofectamine 2000 (Suzhou Beixin Biotechnology Co., Ltd., China). The sequence of siRNA oligos targeting TREM1 was 5′-CCUGGUCUUGGAGUCACUAUCAUAA-3′, and the sequence for control siRNA oligos was 5′-UUAUGAUAGUGACUCCAAGACCAGG-3′. At 48 h after transfection, the transfection efficiency was evaluated by RT-qPCR.

### 2.10. Reverse Transcription and Quantitative PCR (RT-qPCR) Analysis

Using the TRIzol reagent (Invitrogen, Carlsbad, CA, USA), total RNA was extracted from tissues or cells. Reverse transcription was carried out utilizing the PrimeScript® RT Master Mix Perfect Real Time Reagent Kit (Takara Bio Inc., Shiga Prefecture, Japan), and cDNA was used as the template for real-time PCR on an AB7500 RT-PCR instrument (Applied Biosystems, Foster City, CA, USA). The 2^−*ΔΔ*Ct^ method was used to calculate the relative expression levels of the genes after normalization with the internal control GAPDH gene [25]. The sequences of the oligonucleotides used in this study were as follows: TREM1: forward, 5′-AAGTATGCCAGAAGCAGGAAGG-3′, reverse, 5′-GGTAGGGTCATCTTTCAGGGTGT-3′; HMGB1: forward, 5′-ACCCGGATGCTTCTGTCAAC-3′, reverse, 5′-ACAAGAAGGCCGAAGGAGGC-3′; TLR-4: forward, 5′-AGAATGAGGACTGGGTGA-3′, reverse, 5′-AGCGGCTACTCAGAAACT-3′; NF-*κ*B: forward, 5′-GCAAACCTGGGAATACTTCATGTGACTAAG-3′, reverse, 5′-ATAGGCAAGGTCAGAATGCACCAGAAGTCC-3′; TNF-*α*: forward, 5′-TGACAAGCCTGTAGCCCACG-3′, reverse, 5′-TTGTCTTTGATCCATGCCG-3′; IL-6: forward, 5′-GGCCCTTGCTTTCTCTTCG-3′, reverse, 5′-ATAATAAAGTTTTGATTAGT-3′; IL-1*β*: forward, 5′-ATAAGCCCACTCTACACCT-3′, reverse, 5′-ATTGGCCCTGAAAGGAGAGAGA-3′; GAPDH: forward, 5′-CCTCTATGCCAACACAGTGC-3′, reverse, 5′-ACATCTGCTGGAAGGTGGAC-3′.

### 2.11. Western Blot Assay

Fresh tissues and cultured cells were lysed using a radioimmunoprecipitation assay (RIPA) buffer (Beyotime, Shanghai, China) containing a protease inhibitor cocktail. The protein concentrations of the samples were measured by a BCA Protein Assay Kit (Bio-Rad, USA). The lysate was loaded onto 12% (*w*/*v*) sodium dodecyl sulphate-polyacrylamide gel electrophoresis (SDS-PAGE) gels. After electrophoresis, separated strips were transferred onto polyvinylidene fluoride (PVDF) membranes (Millipore, USA) together with a visible prestained protein marker. The membranes were blocked overnight with 5% (*w*/*v*) skim milk in PBS at 4°C and then separately incubated with rabbit anti-HMGB1 monoclonal antibody (1 : 600; ab79823, Abcam, UK), rabbit anti-NF-*κ*B monoclonal antibody (1 : 1000; ab16502), rabbit anti-p-p65 monoclonal antibody (1 : 1000; Cat No. 3033, CST, USA), rabbit anti-p-I*κ*Ba monoclonal antibody (1 : 1000; Cat No. 1241, CST), rabbit anti-TLR-4 polyclonal antibody (1/500; ab13556), rabbit anti-HMBG-1 polyclonal antibody (1/500; ab18256), rabbit anti-NF-*κ*B monoclonal antibody (1 : 1000; ab32360), rabbit anti-GAPDH monoclonal antibody (1 : 1000; Cat No. 6813, CST), and rabbit anti-*β*-actin monoclonal antibody (1 : 1000; Cat No. 4970, CST). After washing with PBST (0.5‰ Tween-20) three times, the membranes were incubated for 1 h with a goat anti-rabbit IgG (H+L) horseradish peroxidase- (HRP-) labeled secondary antibody (1 : 10,000; ab205718). Finally, the blot was developed using enhanced chemiluminescence reagents (CoWin Biotech Co., Ltd., Beijing, China).

### 2.12. Statistical Analysis

All analyses were performed using the GraphPad Prism software. Data are expressed as mean ± standard deviation (SD). One-way analysis of variance (ANOVA) was used for multiple comparisons, followed by Tukey's test. Statistical significance was considered when *p* < 0.05.

## 3. Results

### 3.1. TREM1 Inhibitor LP17 Attenuates the Injury of the Small Intestine Tissues in Rats with LPS-Induced Acute Intestinal Dysfunction

**Figure 1:**
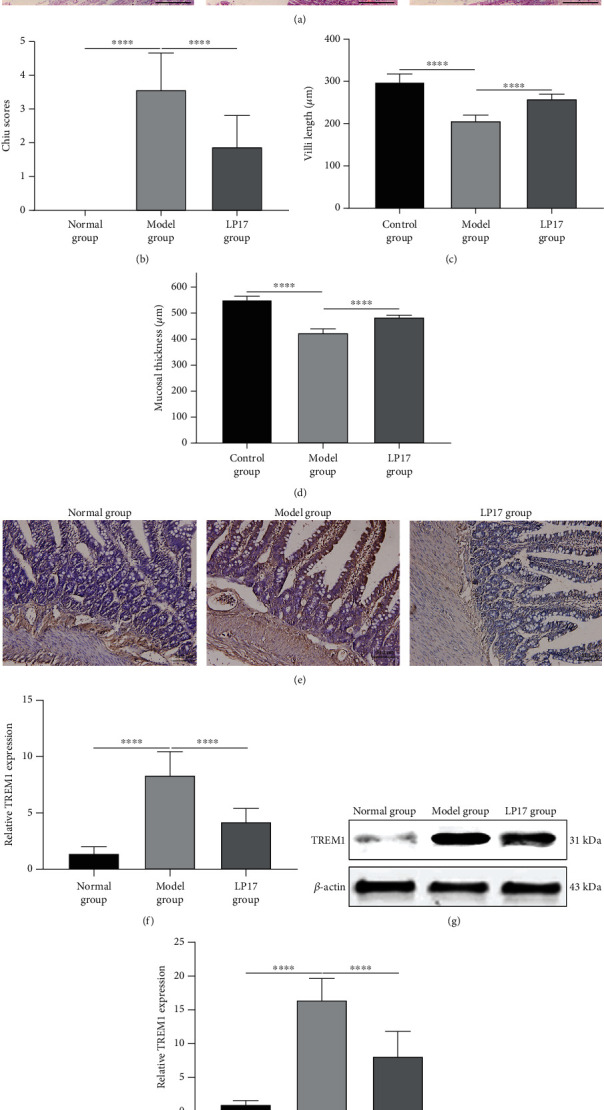
TREM1 inhibitor LP17 attenuates the injury of the small intestine tissues in rats with LPS-induced acute intestinal dysfunction. Rats were randomly assigned into three groups: normal (*n* = 20), model (*n* = 14), and LP17 (*n* = 17). The rats in the model group and the LP17 group received intraperitoneal injection of 4.5 mg/kg LPS. The rats in the LP17 group received LP17 administration (3.5 mg/kg body weight) through the vena caudalis at the time of LPS injection, while the rats in the normal and model groups were injected with equal volume of saline. (a) Representative images of histopathological analysis of the small intestine tissues by H&E staining. Scale bar = 50 *μ*m. (b) The Chiu scores (*F* value = 87.94), (c) length of villi (*F* value = 149.0), and (d) mucosal thickness (*F* value = 453.6) of the small intestine were assessed in the indicated groups. (e, f) Representative images and quantitative results of immunohistochemistry for TREM1 expression (*F* value = 82.45) in the small intestine tissues of the indicated groups. Scale bar = 50 *μ*m. (g, h) Representative images and quantitative results of western blot showing the expression levels of TREM1 and the internal control gene *β*-actin in the rat small intestine tissues (*F* value = 103.4). One-way analysis of variance (ANOVA) was used for multiple comparisons, followed by Tukey's test. ^∗∗∗∗^*p* < 0.0001.

To investigate the potentially protective role of LP17 on acute intestinal dysfunction, we firstly established a 4.5 mg/kg LPS-induced acute intestinal dysfunction rat model. After three days, the mortality of the control, model, and LP17 groups was 0, 30%, and 15%, respectively. Therefore, the number of remaining rats was 20 in the normal group, 14 in the model group, and 17 in the LP17 group, indicating that LP17 could decrease LPS-induced mortality. H&E staining was presented to examine the degree of injury in small intestine tissues from the three groups. As shown in Figure 1(a), the small intestine tissues in the model group displayed mild capillary congestion and denuded villi. Furthermore, they had crypts with exposed lamina propria, dilated and exposed capillaries with evidence of hemorrhage and ulceration, and increased cellularity of the lamina propria. Compared with the model group, LP17 treatment significantly ameliorated LPS-induced intestine injury. In the LP17 group, there was the extended subepithelial space with the epithelial layer lifting up in sheets, the presence of a few denuded villi, crypts with exposed lamina propria, and dilated and exposed capillaries with signs of hemorrhage and increased cellularity of the lamina propria (Figure 1(a)). Moreover, the Chiu score in the LP17 group was significantly lower than that in the model group (Figure 1(b)). Furthermore, compared with the control group, the model group had significantly reduced length of intestinal villi (Figure 1(c)) and thickness of intestinal mucosa (Figure 1(d)). However, LP17 treatment markedly and partially reversed the above changes in LPS-induced acute intestinal dysfunction rats. Since LP17 is a specific inhibitor of TREM1, we set out to determine whether the expression of TREM1 in small intestinal tissues was affected by LPS injection and LP17 administration by immunohistochemistry staining and western blot assays. As expected, the immunohistochemistry staining results demonstrated that the model group had evidently higher expressions of TREM1 than the normal group, while the LP17 group significantly attenuated the increase of TREM1 expression caused by LPS treatment (Figures 1(e) and 1(f)). Furthermore, we found that TREM1 was mainly expressed in the myeloid cells of intestinal tissues. Consistently, the similar results of TREM1 expression in small intestine tissues of rats were also observed by western blot assays (Figures 1(g) and 1(h)). Taken together, these results indicated that TREM1 inhibitor LP17 attenuated the injury of the small intestine tissues in rats with LPS-induced acute intestinal dysfunction.

### 3.2. LP17 Ameliorates Apoptosis of Intestinal Epithelium Cells and Intestinal Mucosal Permeability in LPS-Induced Acute Intestinal Dysfunction Rats

**Figure 2:**
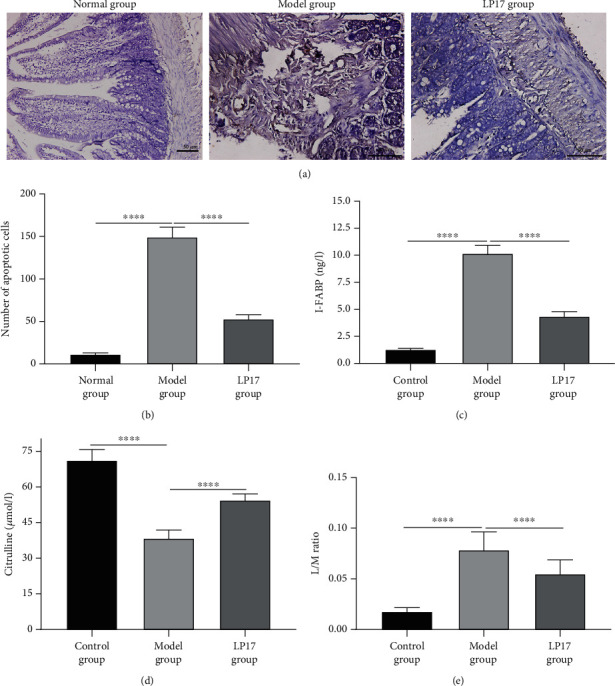
LP17 ameliorates apoptosis of intestinal epithelium cells and intestinal mucosal permeability in LPS-induced acute intestinal dysfunction rats. (a, b) Apoptosis in the small intestine tissues of rats in the indicated groups was determined by TUNEL assay. (a) Representative images of TUNEL staining were shown, and (b) the number of apoptotic cells per each view field was quantified (*F* value = 557.6). Scale bar = 50 *μ*m. (c–e) The levels of (c) I-FABP (*F* value = 1319), (d) citrulline (*F* value = 301.1) in blood samples, and (e) the L/M ratio in the intestinal permeability assay (*F* value = 99.37) were summarized in the indicated groups. *n* = 20 for the normal group; *n* = 14 for the model group; *n* = 17 for the LP17 group. One-way analysis of variance (ANOVA) was used for multiple comparisons, followed by Tukey's test. ^∗∗∗∗^*p* < 0.0001.

The mucosa of the small intestine is lined by a single-layer epithelium. We next conducted the TUNEL assay to observe whether LPS injection and LP17 administration affected apoptosis of intestinal epithelium tissues. Compared with the normal group, the model group had a significantly higher number of apoptotic cells in the intestinal epithelium (Figures 2(a) and 2(b)). However, administration of LP17 remarkably reduced the number of apoptotic cells induced by LPS (Figures 2(a) and 2(b)). Elevated I-FABP and decreased citrulline levels are the indicators of enterocyte mass damage [11, 12]. As shown in Figure 2(c), the expression of I-FABP in the model group was significantly higher than that in the control group. In addition, the model group showed a decreased level of citrulline in comparison to the control group (Figure 2(d)). However, LP17 administration caused a significant decrease in the I-FABP level (Figure 2(c)) and a remarkable increase in the citrulline level (Figure 2(d)) in LPS-induced rats. The tight junctions between cells are destroyed by the apoptosis of epithelial cells, thereby increasing the intestinal permeability [37]. We found that the intestinal permeability in LPS-induced rats was notably higher than in controls, as assessed by the L/M ratio (Figure 2(e)). LP17 administration resulted in a clear attenuation in the mucosal permeability damage compared with the model group (Figure 2(e)). Collectively, our results suggested that LP17 could markedly ameliorate the apoptosis of the small intestine tissues in LPS-induced acute intestinal dysfunction rats and preserve the intestinal mucosal permeability.

### 3.3. LP17 Reduces Intestinal and Systemic Inflammatory Response and Activation of the NF-*κ*B Signaling in LPS-Induced Acute Intestinal Dysfunction Rats

**Figure 3:**
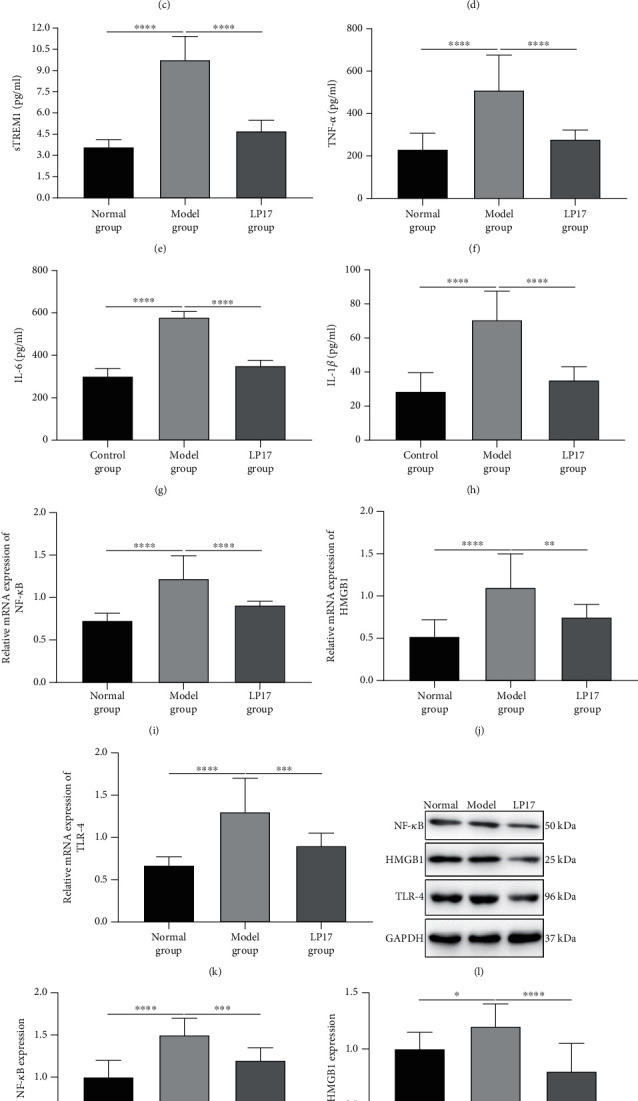
LP17 reduces inflammatory response and activation of the NF-*κ*B signaling in LPS-induced acute intestinal dysfunction rats. (a–d) The levels of (a) soluble TREM1 (sTREM1; *F* value = 40.98), (b) TNF-*α* (*F* value = 39.30), (c) IL-6 (*F* value = 231.5), and (d) IL-1*β* (*F* value = 186.3) in the plasma samples of rats from the indicated groups at 36 h after LPS treatment for 3 days were measured by ELISA. (e–h) The levels of (e) sTREM1 (*F* value = 121.6), (f) TNF-*α* (*F* value = 26.85), (g) IL-6 (*F* value = 310.5), and (h) IL-1*β* (*F* value = 44.45) in the intestine tissue samples of rats from the indicated groups at 36 h after LPS treatment for 3 days were measured by ELISA. (i–k) The mRNA levels of (i) NF-*κ*B (*F* value = 42.46), (j) HMGB1 (*F* value = 16.10), and (k) TLR-4 (*F* value = 22.17) in the small intestine tissues of rats from the indicated groups at 36 h after LPS treatments for 3 days were determined by RT-qPCR. *n* = 20 for the normal group; *n* = 14 for the model group; *n* = 17 for the LP17 group. (l) Representative images of western blot results for NF-*κ*B, HMGB1, and TLR-4 in the small intestine tissues of rats from the indicated groups. (m–o) The expression levels of (m) NF-*κ*B (*F* value = 25.95), (n) HMGB1 (*F* value = 13.44), and (o) TLR-4 (*F* value = 360.2) proteins were quantified according to the western blot results. One-way analysis of variance (ANOVA) was used for multiple comparisons, followed by Tukey's test. ^∗^*p* < 0.05; ^∗∗^*p* < 0.01; ^∗∗∗^*p* < 0.001; ^∗∗∗∗^*p* < 0.0001.

We further investigated whether modulation of the TREM1 pathway influenced the systemic inflammatory response in the LPS-induced acute intestinal dysfunction rats. Compared with the normal group, the model group had significantly increased plasma concentrations of sTREM1 (Figure 3(a)), TNF-*α* (Figure 3(b)), IL-6 (Figure 3(c)), and IL-1*β* (Figure 3(d)). However, LP17 administration distinctly reduced the levels of these inflammatory cytokines in plasma of LPS-induced acute intestinal dysfunction rats (Figures 3(a)–3(d)). These inflammatory cytokines were also measured in the intestine tissues. The results showed that sTREM1 (Figure 3(e)), TNF-*α* (Figure 3(f)), IL-6 (Figure 3(g)), and IL-1*β* (Figure 3(h)) levels were significantly increased in the intestine tissues of the model group in comparison to the normal group. After treatment with LP17, their levels were markedly reduced in the intestine tissues of the LPS-induced acute intestinal dysfunction rats (Figures 3(e)–3(h)). Moreover, we determined the mRNA expression levels of NF-*κ*B, HMGB1, and TLR-4 in the small intestine mucosa tissues by RT-qPCR. Our results showed that there were significantly higher expression levels of NF-*κ*B (Figure 3(i)), HMGB1 (Figure 3(j)), and TLR-4 (Figure 3(k)) in the model group than the control group. LP17 administration distinctly decreased their mRNA expression levels in the model group (Figures 3(i)–3(k)). Western blot was also presented to detect their expression at the protein levels (Figure 3(l)). Consistent with our RT-qPCR results, the expression levels of NF-*κ*B (Figure 3(m)), HMGB1 (Figure 3(n)), and TLR-4 (Figure 3(o)) proteins were significantly increased in the model group compared to the control group. Under the administration of LP17, their expression levels were distinctly reduced in the small intestine mucosa tissues of LPS-induced acute intestinal dysfunction rats (Figures 3(m)–3(o)).

### 3.4. Knockdown of TREM1 Suppresses Inflammatory Response and NF-*κ*B Signaling Activation in LPS-Induced Intestinal Epithelium Cells

**Figure 4:**
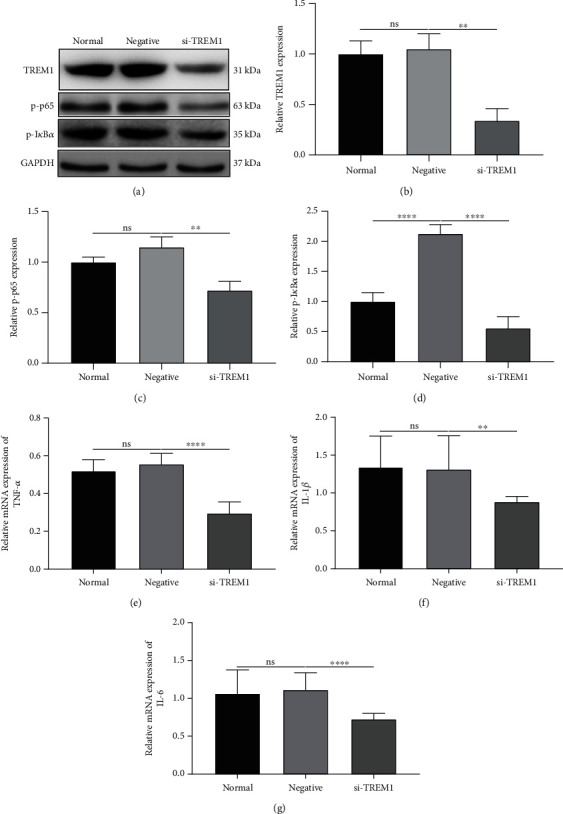
Knockdown of TREM1 suppresses the inflammatory response and NF-*κ*B signaling activation in LPS-induced intestinal epithelium cells. (a) Representative images of western blot results for TREM1, p-p65, ad p-I*κ*Ba in LPS-induced intestinal epithelium-6 (IEC-6) cells transfected with TREM1 specific siRNA oligos or negative control oligos. (b–d) The expression levels of (b) TREM1 (*F* value = 26.27), (c) p-p65 (*F* value = 20.81), and (d) p-I*κ*Ba (*F* value = 72.79) were quantified in the indicated groups. (e–g) The mRNA expression levels of (e) TNF-*α* (*F* value = 89.24), (f) IL-6 (*F* value = 14.15), and (g) IL-1*β* (*F* value = 9.290) were evaluated by RT-qPCR. *n* = 3 for each group. One-way analysis of variance (ANOVA) was used for multiple comparisons, followed by Tukey's test. ns: no statistical significance; ^∗∗^*p* < 0.01; ^∗∗∗∗^*p* < 0.0001.

To substantiate the role of TREM1 in regulating inflammatory response during LPS-induced acute intestinal dysfunction, we silenced TREM1 in LPS-stimulated rat intestinal epithelium cell line IEC6. Our western blot results confirmed that TREM1 was successfully silenced in LPS-induced IEC6 cells (Figures 4(a) and 4(b)). Furthermore, we assessed the effects of TREM1 knockdown on NF-*κ*B signaling. The results showed that compared with the negative siRNA oligo-transfected group, the knockdown of TREM1 markedly reduced protein expressions of p-p65 (Figure 4(c)) and p-I*κ*B*α* (Figure 4(d)), two critical components of the NF-*κ*B signaling pathway. Moreover, silencing TREM1 significantly reduced the mRNA expressions of TNF-*α* (Figure 4(e)), IL-1*β* (Figure 4(f)), and IL-6 (Figure 4(g)) in LPS-induced IEC6 cells. Thus, knockdown of TREM1 could suppress inflammatory response in LPS-induced intestinal epithelium cells, which might be related to the NF-*κ*B signaling.

## 4. Discussion

In this study, we observed the protective effects of TREM1 inhibitor LP17 against LPS-induced acute intestinal dysfunction, which was associated with inhibition of apoptosis of intestinal epithelium cells and intestinal and systemic inflammatory responses. Mechanically, LP17 could inactivate the NF-*κ*B signaling in LPS-induced intestinal tissues and cells.

In the current study, we constructed an LPS-induced acute intestinal dysfunction rat model. The citrulline levels in the model group were notably lower than those in the normal group, confirming that the model was successfully established. TREM1 has a major function of triggering the inflammatory response [15, 22, 32]. It can induce cytokine production through binding with its adaptor molecule, TYRO protein tyrosine kinase-binding protein (TYROBP) [14, 15, 39]. In addition, TREM1 can synergize with various TLRs, causing amplification of the inflammatory response [12, 14, 15, 22, 39, 40]. Herein, we found that the expression of TREM1 was significantly elevated in LPS-induced small intestine tissues. As in previous studies, mRNA expression levels of TREM1 were negatively correlated with plasma citrulline levels [25]. Moreover, sTREM1 was distinctly upregulated in the serum of the model group in comparison to the normal group, indicating that TREM1 could be involved in the occurrence and development of LPS-induced acute intestinal dysfunction. It has been reported that the survival of LPS-induced septic shock mice can be prolonged by blocking TREM1 [26]. Wang et al. revealed that TREM1 blockade could protect animals from death caused by Pseudomonas aeruginosa-induced sepsis [39]. In this study, our results demonstrated that LP17 could markedly decrease the mortality in mice with LPS-induced acute intestinal dysfunction. Furthermore, we investigated the effects of LP17 on intestinal permeability, intestinal damage, and inflammatory response in LPS-induced rat models. Overall, model rats exhibited increased intestinal permeability and systemic and intestinal inflammation, which were ameliorated by administration of TREM1 inhibitor LP17. Also, LP17 treatment reduced the expression levels of I-FABP and increased the citrulline levels in rats with LPS-induced acute intestinal dysfunction. Partially consistent with a previous study, intestinal mucosa barrier damage in pancreatitis-associated inflammatory bowel disease models could be ameliorated by TREM1 blockade LP17 treatment [22]. These findings implied that LP17 treatment improved the intestinal barrier function and intestinal mucosa damage induced by LPS.

It has been reported that TREM1 is upregulated in neutrophils both from sepsis patients and LPS-induced septic shock mice [41]. Activation of TREM1 together with stimulation by TLR ligands such as LPS causes increased TNF-*α* production [42]. The activation of TLRs is closely related to TREM1 activation, and both of them act synergistically to increase inflammation [43]. Furthermore, multiple ligands, such as HMGB1 and HSP70, can activate TREM1 [44, 45]. MGB1 released from macrophages can serve as a sepsis biomarker [46]. HMGB1 is a ligand of TREM1 and TLRs [47]. Previous studies have implied that increased HMGB1 levels activate TREM1 and TLR downstream signaling, leading to an increase in the secretion of inflammatory cytokines, such as IL-6, TNF-*α*, and IL-1*β* [15]. Therefore, inhibition of TREM1 can improve the inflammatory state in sepsis. To further explore the underlying molecular mechanism of LP17 on treating LPS-induced acute intestinal dysfunction, we evaluated the mRNA and protein levels of HMGB1, TLR-4, and NF-*κ*B in small intestine tissues. The results demonstrated that LP17 distinctly reduced their expression in LPS-induced small intestine tissues. In agreement with previous studies, LP17 inhibited the LPS-stimulated increase in the levels of sTREM1, TNF-*α*, IL-6, and IL-1*β* in plasma and intestinal samples, indicating the suppression of intestinal and systemic inflammatory responses. Moreover, our results suggested that TREM1 knockdown protected against LPS-induced acute intestinal dysfunction in vitro.

Our study revealed that TREM1 could become a potential therapeutic target in LPS-induced acute intestinal dysfunction. However, undoubtedly, since the LPS-induced model may not fully represent the various types of acute intestinal dysfunction encountered in most clinical settings, and we only focused on one time point of LPS-treated intestinal injury in rats, more preclinical work should be required to consolidate the clinical application concerning TREM1 target therapy for acute intestinal dysfunction.

## 5. Conclusion

Taken together, our findings revealed that TREM1 inhibitor LP17 ameliorated LPS-induced acute intestinal dysfunction, which could be related to the inhibition of the TREM1-HMGB1-TLR-4-NF-*κ*B signal transduction pathway as well as the downstream production of proinflammatory cytokines. These findings demonstrated that TREM1 inhibition protected rat intestinal tissues from hyperinflammatory injury in response to endotoxin. In addition, these results also indicated that TREM1 can be used as an effective therapeutic target in LPS-induced acute intestinal dysfunction. Our study could shed a new light on the development of orally bioavailable formulation of LP17 in treating patients with acute intestinal dysfunction.

## Data Availability

The data used to support the findings of this study are available from the corresponding author upon request.

## Abbreviations

TREM1: Triggering receptor expressed on myeloid cells-1
LPS: Lipopolysaccharide
L/M: Lactulose/mannitol
sTREM1: Soluble TREM1
HMGB1: High-mobility group box 1
TLR-4: Toll-like receptor 4
IEC-6: Intestinal epithelium-6
TNF: Tumor necrosis factor
IL: Interleukin
ELISA: Enzyme-linked immunosorbent assay
H&E: Hematoxylin-eosin
RT-qPCR: Reverse transcription and quantitative PCR.

## References

[1] Zhang L., Wei X., Zhang R. (2019). A novel peptide ameliorates LPS-induced intestinal inflammation and mucosal barrier damage via its antioxidant and antiendotoxin effects. *International Journal of Molecular Sciences*.

[2] Li J., Wang H., Zheng Z. (2018). Mkp-1 cross-talks with Nrf2/Ho-1 pathway protecting against intestinal inflammation. *Free Radical Biology & Medicine*.

[3] de Souza H. S., Fiocchi C. (2016). Immunopathogenesis of IBD: current state of the art. *Nature Reviews. Gastroenterology & Hepatology*.

[4] Meng J.-b., Jiao Y.-n., Zhang G. (2018). Electroacupuncture improves intestinal dysfunction in septic patients: a randomised controlled trial. *BioMed Research International*.

[5] Mittal R., Coopersmith C. M. (2014). Redefining the gut as the motor of critical illness. *Trends in Molecular Medicine*.

[6] Puleo F., Arvanitakis M., Van Gossum A., Preiser J. C. (2011). Gut failure in the ICU. *Seminars in Respiratory and Critical Care Medicine*.

[7] Piton G. B. F., Cypriani B. (2013). Enterocyte damage in critically ill patients is associated with shock condition and 28-day mortality. *Critical Care Medicine*.

[8] Shen L. J., Guan Y. Y., Wu X. P. (2015). Serum citrulline as a diagnostic marker of sepsis-induced intestinal dysfunction. *Clinics and Research in Hepatology and Gastroenterology*.

[9] Hotchkiss R. S., Monneret G., Payen D. (2013). Sepsis-induced immunosuppression: from cellular dysfunctions to immunotherapy. *Nature Reviews. Immunology*.

[10] Singer M., Deutschman C. S., Seymour C. W. (2016). The third international consensus definitions for sepsis and septic shock (sepsis-3). *JAMA*.

[11] Shi X., Zhang Y., Wang H., Zeng S. (2017). Effect of triggering receptor expressed on myeloid cells 1 (TREM-1) blockade in rats with cecal ligation and puncture (CLP)-induced sepsis. *Medical Science Monitor*.

[12] Bleharski J. R., Kiessler V., Buonsanti C. (2003). A role for triggering receptor expressed on myeloid cells-1 in host defense during the early-induced and adaptive phases of the immune response. *Journal of Immunology*.

[13] Liu M., Zhang Y., Xiong J. Y., Wang Y., Lv S. (2016). Etomidate mitigates lipopolysaccharide-induced CD14 and TREM-1 expression, NF-*κ*B activation, and pro-inflammatory cytokine production in rat macrophages. *Inflammation*.

[14] Sharif O., Knapp S. (2008). From expression to signaling: roles of TREM-1 and TREM-2 in innate immunity and bacterial infection. *Immunobiology*.

[15] Arts R. J., Joosten L. A., van der Meer J. W., Netea M. G. (2013). TREM-1: intracellular signaling pathways and interaction with pattern recognition receptors. *Journal of Leukocyte Biology*.

[16] Brenner T., Uhle F., Fleming T. (2016). Soluble TREM-1 as a diagnostic and prognostic biomarker in patients with septic shock: an observational clinical study. *Biomarkers*.

[17] Qian L., Weng X. W., Chen W., Sun C. H., Wu J. (2014). TREM-1 as a potential therapeutic target in neonatal sepsis. *International Journal of Clinical and Experimental Medicine*.

[18] Schenk M., Bouchon A., Seibold F., Mueller C. (2007). TREM-1--expressing intestinal macrophages crucially amplify chronic inflammation in experimental colitis and inflammatory bowel diseases. *The Journal of Clinical Investigation*.

[19] Natale G., Biagioni F., Busceti C. L., Gambardella S., Limanaqi F., Fornai F. (2019). TREM receptors connecting bowel inflammation to neurodegenerative disorders. *Cell*.

[20] Yang F. C., Chiu P. Y., Chen Y., Mak T. W., Chen N. J. (2019). TREM-1-dependent M1 macrophage polarization restores intestinal epithelium damaged by DSS-induced colitis by activating IL-22-producing innate lymphoid cells. *Journal of Biomedical Science*.

[21] Denning N. L., Aziz M., Ochani M., Prince J. M., Wang P. (2020). Inhibition of a triggering receptor expressed on myeloid cells-1 (TREM-1) with an extracellular cold-inducible RNA-binding protein (eCIRP)-derived peptide protects mice from intestinal ischemia-reperfusion injury. *Surgery*.

[22] Dang S., Shen Y., Yin K., Zhang J. (2012). TREM-1 promotes pancreatitis-associated intestinal barrier dysfunction. *Gastroenterology Research and Practice*.

[23] He C., Deng J., Hu X. (2019). Vitamin A inhibits the action of LPS on the intestinal epithelial barrier function and tight junction proteins. *Food & Function*.

[24] Chen M., Liu Y., Xiong S. (2019). Dietary l-tryptophan alleviated LPS-induced intestinal barrier injury by regulating tight junctions in a Caco-2 cell monolayer model. *Food & Function*.

[25] Yuan Z., Syed M., Panchal D. (2016). TREM-1-accentuated lung injury via miR-155 is inhibited by LP17 nanomedicine. *Lung cellular and molecular physiology*.

[26] Bouchon A., Facchetti F., Weigand M. A., Colonna M. (2001). TREM-1 amplifies inflammation and is a crucial mediator of septic shock. *Nature*.

[27] Zhuang S., Zhong J., Bian Y. (2019). Rhein ameliorates lipopolysaccharide-induced intestinal barrier injury via modulation of Nrf2 and MAPKs. *Life Sciences*.

[28] Gil-Cardoso K., Comitato R., Ginés I. (2019). Protective effect of proanthocyanidins in a rat model of mild intestinal inflammation and impaired intestinal permeability induced by LPS. *Molecular Nutrition & Food Research*.

[29] Unno N., Wang H., Menconi M. J. (1997). Inhibition of inducible nitric oxide synthase ameliorates endotoxin- induced gut mucosal barrier dysfunction in rats. *Gastroenterology*.

[30] Chang J. X., Chen S., Ma L. P. (2005). Functional and morphological changes of the gut barrier during the restitution process after hemorrhagic shock. *World Journal of Gastroenterology*.

[31] Crenn P., Messing B., Cynober L. (2008). Citrulline as a biomarker of intestinal failure due to enterocyte mass reduction. *Clinical Nutrition*.

[32] Yuan B., Zhou S., Lu Y. (2015). Changes in the expression and distribution of claudins, increased epithelial apoptosis, and a mannan-binding lectin-associated immune response lead to barrier dysfunction in dextran sodium sulfate-induced rat colitis. *Gut and liver*.

[33] Vyas D., Robertson C. M., Stromberg P. E. (2007). Epithelial apoptosis in mechanistically distinct methods of injury in the murine small intestine. *Histology and Histopathology*.

[34] Chiu C.-J. (1970). Intestinal mucosal lesion in low-flow states. I. A morphological, hemodynamic, and metabolic reappraisal. *Archives of Surgery*.

[35] Abdel-Aziz H., Schneider M., Neuhuber W. (2016). GPR84 and TREM-1 signaling contribute to the pathogenesis of reflux esophagitis. *Molecular Medicine*.

[36] Truong A. D., Hong Y., Hoang C. T., Lee J., Hong Y. H. (2017). Chicken IL-26 regulates immune responses through the JAK/STAT and NF-*κ*B signaling pathways. *Developmental and Comparative Immunology*.

[37] Song D., Zong X., Zhang H. (2015). Antimicrobial peptide cathelicidin-BF prevents intestinal barrier dysfunction in a mouse model of endotoxemia. *International Immunopharmacology*.

[38] He S., Guo Y., Zhao J. (2019). Ferulic acid protects against heat stress-induced intestinal epithelial barrier dysfunction in IEC-6 cells via the PI3K/Akt-mediated Nrf2/HO-1 signaling pathway. *International Journal of Hyperthermia*.

[39] Colonna M. (2003). TREMs in the immune system and beyond. *Nature Reviews. Immunology*.

[40] Wang F., Liu S., Wu S. (2012). Blocking TREM-1 signaling prolongs survival of mice with Pseudomonas aeruginosa induced sepsis. *Cellular Immunology*.

[41] Bouchon A., Dietrich J., Colonna M. (2000). Cutting edge: inflammatory responses can be triggered by TREM-1, a novel receptor expressed on neutrophils and monocytes. *Journal of Immunology*.

[42] Tessarz A. S., Weiler S., Zanzinger K., Angelisová P., Horejsí V., Cerwenka A. (2007). Non-T cell activation linker (NTAL) negatively regulates TREM-1/DAP12-induced inflammatory cytokine production in myeloid cells. *Journal of Immunology*.

[43] Fortin C. F., Lesur O., Fulop T. (2007). Effects of TREM-1 activation in human neutrophils: activation of signaling pathways, recruitment into lipid rafts and association with TLR4. *International Immunology*.

[44] Wu J., Li J., Salcedo R., Mivechi N. F., Trinchieri G., Horuzsko A. (2012). The proinflammatory myeloid cell receptor TREM-1 controls Kupffer cell activation and development of hepatocellular carcinoma. *Cancer Research*.

[45] El Mezayen R., El Gazzar M., Seeds M. C., McCall C. E., Dreskin S. C., Nicolls M. R. (2007). Endogenous signals released from necrotic cells augment inflammatory responses to bacterial endotoxin. *Immunology Letters*.

[46] Gámez-Díaz L. Y., Enriquez L. E., Matute J. D. (2011). Diagnostic accuracy of HMGB-1, sTREM-1, and CD64 as markers of sepsis in patients recently admitted to the emergency department. *Academic Emergency Medicine : Official Journal of the Society for Academic Emergency Medicine*.

[47] Subramanian S., Pallati P. K., Sharma P., Agrawal D. K., Nandipati K. C. (2017). Significant association of TREM-1 with HMGB1, TLRs and RAGE in the pathogenesis of insulin resistance in obese diabetic populations. *American Journal of Translational Research*.

